# Optbayesexpt: Sequential Bayesian Experiment Design for Adaptive Measurements

**DOI:** 10.6028/jres.126.002

**Published:** 2021-02-03

**Authors:** Robert D. McMichael, Sean M. Blakley, Sergey Dushenko

**Affiliations:** 1 National Institute of Standards and Technology, Gaithersburg, MD 20899, USA; 2 Institute for Research in Electronics and Applied Physics, University of Maryland, College Park, MD 20742 USA

**Keywords:** automation, Bayesian, experiment design, measurement, parameter estimation, particle flter, python, sequential Monte Carlo

**Software DOI:** https://doi.org/10.18434/M32230

## 1 Summary

Optbayesexpt is a public domain, open-source python package that provides adaptive algorithms for efficient estimation/measurement of parameters in a model function. Parameter estimation is the type of measurement one would conventionally tackle with a sequence of data acquisition steps followed by fitting. The software is designed to provide data-based control of experiments, effectively learning from incoming measurement results and using that information to select future measurement settings live and online as measurements progress. The settings are chosen to have the best chances of improving the measurement results. With these methods optbayesexpt is designed to increase the efficiency of a sequence of measurements, yielding better results and/or lower cost. In a recent experiment, optbayesexpt yielded an order of magnitude increase in speed for measurement of a few narrow peaks in a broad spectral range [1].

**Fig. 1:**
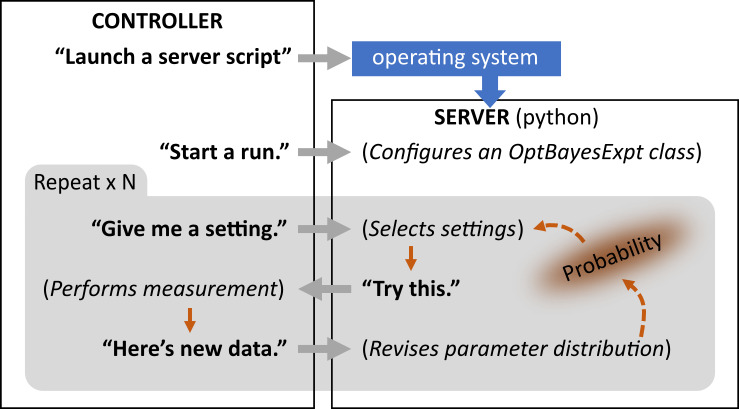
Flowchart showing how optbayesexpt works with a user’s instrumentation program. The instrumentation program (left) starts a python script (right) that runs in the background as a server. The instrumentation program issues a command to set up for a run by creating an OptBayesExpt object. The program then enters a measurement loop, asking for recommended settings, and reporting measurement data. The optbayesexpt server refines the parameter distribution using Bayesian inference and selects high-utility settings “live” based on the accumulated measurement data. The instrumentation program can be written in any language capable of TCP socket communication.

Figure 1 illustrates a possible use scenario where an instrument control program interacts with optbayesexpt functions in a python server script. The server script runs in the background, and the two programs communicate via TCP sockets. For each of *N* measurements, the controller program prompts the server script to recommend high-utility settings based on the parameter probability distribution. The controller then makes measurements and reports the new data back to the server script, which uses Bayesian inference to refine the parameter distribution for use in the next iteration.

The value of optbayesexpt involves a tradeoff between measurement cost and computation cost, so the most promising applications are those where data is expensive and computation is cheap. However, the efficiency of a pre-existing conventional measurement is also an important consideration. If an existing measurement protocol has been heavily optimized, there might be room for only small efficiency gains with optbayesexpt.

The intent of optbayesexpt is to provide sequential Bayesian experiment design tools to statistics non-specialists. The project adopts a “runs good” philosophy:

• If it’s a struggle to use, it can’t run good.
• If technical jargon is a barrier, it can’t run good.
• If the user finds it useful, it runs good.
• If it runs good, it is good.

## 2 Background

This section gives a brief overview of Bayesian experiment design, with citations to selected literature. For a more thorough introduction to the methods, the interested reader is referred to the project manual and to the supplemental information for ref. [1].

The methods implemented in this software are known by several names, including “sequential Bayesian experiment design,” “optimal Bayesian experimental design,” and “Bayesian optimal design.” A thorough review of Bayesian experiment design would include examples from medicine, biology, geology, astrophysics, social sciences, physics and more. Despite this broad applicability, the available software has been fragmented, and has required statistical expertise.

The methods have their roots in the work of Bayes [2], Laplace [3]. A 1995 review article by Chaloner and Verdinelli [4] describes developments that included information theory and decision theory to choose measurement settings with high *utility*. Most commonly, utility is defined as the average predicted decrease in the information entropy of the parameter distribution [5].

While the core principles of sequential Bayesian experimental design are well-established, recent progress has focused on computation. A recent review article by Ryan [6] provides an overview of computational methods. The representation of the parameter probability density is particularly important for Bayesian methods. Approximate Bayes computing (ABC), Markov chain Monte Carlo (MCMC) and sequential Monte Carlo (SMC) are three alternatives. The ideas and algorithms implemented in optbayesexpt are largely drawn from Huan and Marzouk [7] and Granade [8].

Optbayesexpt uses sequential Monte Carlo (SMC) methods to represent the parameter distribution. First introduced in 1993 as a bootstrap filter, [9] and also known as particle filters [10] or swarm filters, SMC methods are used in many diverse fields. SMC methods represent a distribution as a collection of random samples, like points in parameter space, each with an associated weight. A key advantage of this representation is its flexibility: probability density is represented by a combination of particle density and particle weights. Probability density can be directly adjusted through weights, and a *resampling* step ensures efficient computation, essentially by reassigning computational resources from low-weight particles to high-probability regions of parameter space.

## 3 Requirements

The hardware requirements for optbayesexpt are met by many modern desktop and laptop computers capable of running Python 3.x. The demo programs have been tested on a variety of desktops and laptops with Windows^1^1Certain commercial equipment, instruments, or materials are identified in this paper to foster understanding. Such identification does not imply recommendation or endorsement by the National Institute of Standards and Technology, nor does it imply that the materials or equipment identified are necessarily the best available for the purpose. and Linux operating systems.

**Package:** Optbayesexpt requires numpy and scipy packages for computation.

**Package:** Sample scripts require matplotlib for plotting.

**Setup:** A parametric model of the measurement. The model is analogous to a ”ft function” that would be used with least-squares regression. It is important that the model is able to reproduce all of the features that are likely to appear in measurement data. Optbayesexpt used a model with 7 parameters in reference [1].

**Setup:** Arrays of allowed experimental setting values.

**Setup:** Parameter values as arrays of random draws from the *prior* parameter distribution. Parameters correspond to the unknowns of a least-squares ft.

**Input:** Measurement values with corresponding measurement settings.

Some of these requirements are intentionally vague. The measurement in the cycle depicted in Fig. 1 could be as simple as recording a single value, or more complex like an average of repeated measurements or like a sequence of values corresponding to a swept setting value.

## 4 Software Specifications

**Table:** 

**NIST Operating Unit**	Physical Measurement Laboratory
**Category**	Experiment design, Adaptive measurement
**Targeted Users**	Experimenters, measurement automators
**Operating Systems**	Windows, Mac, Linux. Any python compatible OS.
**Programming Language**	Python 3.x +
**Inputs/Outputs**	Setup: model function, allowed settings and initial parameter distributions Inputs: measurement data with corresponding settings Outputs: settings, posterior parameter distribution and statistics
**Documentation**	https://pages.nist.gov/optbayesexpt
**Accessibility**	Public
**Disclaimer**	https://www.nist.gov/director/licensing

## 5 Methods for Validation

The package is provided with a suite of unit tests that check the basic functionality of the software. Also included is an inference test using a trivial measurement simulation that checks that the true value falls within the distribution’s 95% credible range approximately 95 out of 100 times. Optbayesexpt is also provided with a suite of demonstration simulations that can be run to check functionality.

The experiment model and parameter distribution form the core of the calculations, and both are under the user’s direct control, so reliability, numerical stability and speed have not been thoroughly tested. We strongly urge users to test their own programs using measurement simulations. In simulation, the user can compare results of measurement runs with known, true model parameter values and verify the reliability and speed of the program. The package includes a MeasurementSimulator() method to facilitate simulation.

## 6 Behavior Monitoring

A good way to monitor a run (real or simulated) is to follow a standard deviation of the parameter distribution. Typical behavior during a run involves three phases. In the first phase, the standard deviations fluctuate, but remain close to initial values. In a second phase, standard deviations drop rapidly as the algorithm focuses on a neighborhood of high-probability parameters and rules out other regions of parameter space. Although the general trend in this phase is a rapid decrease, the standard deviation tends to fluctuate dramatically. In the third phase, the standard deviations exhibit a smoother 1*/^√^N* behavior with iteration count *N*. In this stage, a few choice settings tend to be repeated as the system suppresses noise through averaging.

In the third phase, a sudden drop in a standard deviation may be a sign of particle impoverishment [11]. This condition is a recognized pitfall of SMC probability distribution methods having too few particles. Low probability regions can become completely empty during resampling, effectively eliminating those parameter combinations from further consideration. Simulations show that particle impoverishment causes errors, i.e. in parameter distributions that disagree with the true values. The remedy for particle impoverishment is to increase the number of particles in the distribution.

Optbayesexpt comes with no promise of accurate or reliable results. It is the user’s responsibility to verify the quality of their own results. Modeling with tests of different particle numbers and comparing results with known parameter values is highly recommended.
